# The Art of Sleeve Gastrectomy

**DOI:** 10.3390/jcm13071954

**Published:** 2024-03-28

**Authors:** Shahed Tish, Ricard Corcelles

**Affiliations:** Bariatric and Metabolic Institute, Department of General Surgery, Cleveland Clinic, Cleveland, OH 44195, USA; corcelr@ccf.org

**Keywords:** sleeve gastrectomy, SG, LSG, metabolic and bariatric surgery

## Abstract

Sleeve gastrectomy (SG) has historically evolved from gastroplasty and anti-reflux procedures into one of the most commonly performed primary metabolic surgeries in the United States and worldwide. Initially initiated in the 1980s as part of the duodenal switch procedure, its standalone effectiveness and simplicity have led to increasing popularity globally. The rise in obesity rates transcends age boundaries, alarmingly affecting not only adults but also the younger demographic. This escalating trend is concerning, as it predisposes these populations to numerous future health complications, as well as highlighting the critical necessity for a safe and potent weight loss strategy. Although sleeve gastrectomy carries a higher risk for gastroesophageal reflux disease (GERD) compared to other bariatric procedures, it stands out as a reliable, safe and effective surgical solution for obesity. It is particularly beneficial for adolescents and patients with complex medical comorbidities, including, but not limited to, heart failure and immunocompromisation. It has also served as a bridge for transplants in morbidly obese patients with end-stage heart, liver and kidney disease due to its favorable safety profile.

## 1. Introduction

**Figure 1:**
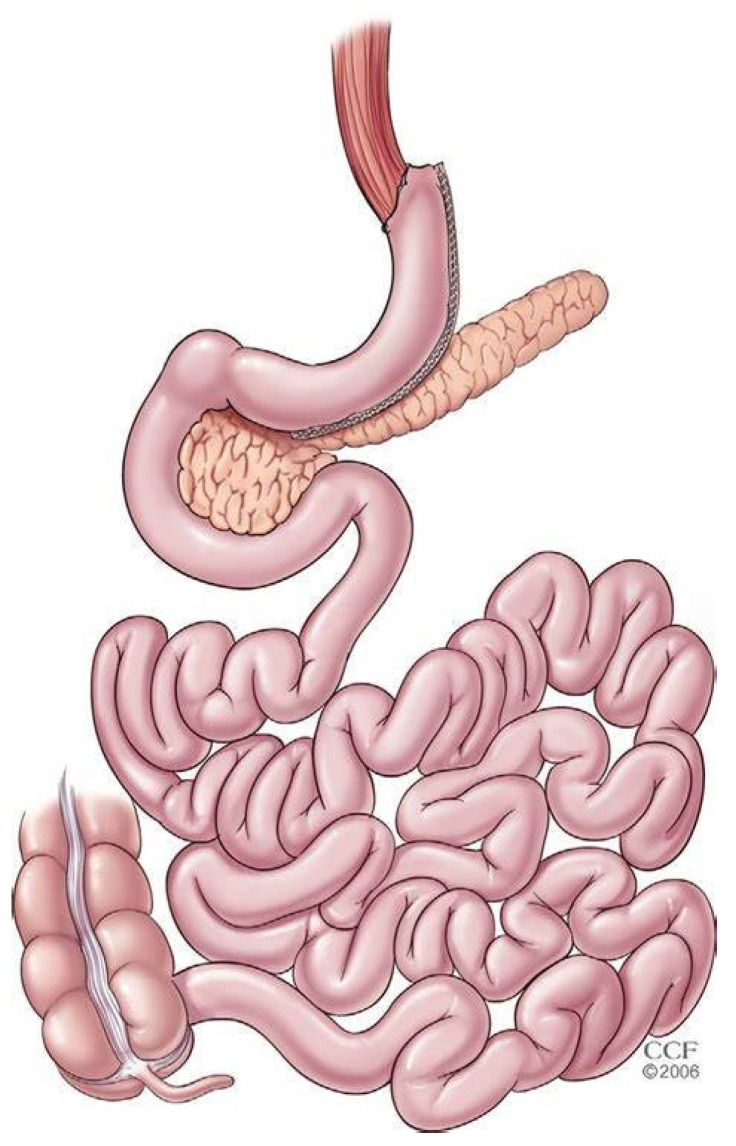
Surgical anatomy of sleeve gastrectomy.

Sleeve gastrectomy (SG) is an evolution rather than the creation of a standalone procedure (Figure 1). Its roots can be traced back to earlier gastroplasty procedures as part of anti-reflux surgeries [1,2]. The modern concept of sleeve gastrectomy began to take shape in the late 1980s. The first known procedure of this kind was performed by Dr. Doug Hess in Bowling Green, Ohio, in March 1988, as part of the broader duodenal switch operation [3]. Initially, this procedure was not intended as a standalone treatment but as a component of a more complex surgery. However, over time, the sleeve gastrectomy evolved into its own entity, characterized by its effectiveness in weight loss and safety profile [4]. In 1993, Marceau et al. published their experience altering the gastrectomy portion of the biliopancreatic diversion procedure. In an attempt to preserve vagal continuity, the distal gastrectomy was replaced by a parietal gastrectomy along the greater curvature of the stomach [5]. The biliopancreatic diversion procedure soon evolved into its laparoscopic version. This operation included the creation of a 150–200 mL sleeve gastrectomy to be anastomosed with the distal ileum [6]. In 2004, Almogy et al. suggested longitudinal gastrectomy (LG) as a safe alternative to duodenal switch, which was thought to be the most effective weight loss operation at that time, in high-risk patients, classified as patients with a body mass index (BMI) > 50 kg/m^2^ [7]. The procedure’s rise in popularity is attributed to its relative simplicity compared to other bariatric surgeries and its effectiveness in significant weight loss. Per the International Federation for the Surgery of Obesity and Metabolic Disorders’ (IFSO) 8th report, published in 2023, SG is currently the most common bariatric procedure performed globally (60.4% of all metabolic procedures) and in the United States (68.8% of all metabolic procedures) [8].

## 2. Epidemiology of Obesity

The global challenge of obesity and its consequent strain on individuals and healthcare systems is escalating. According to the World Health Organization (WHO), roughly 16% of the global adult population were classified as obese in 2022. This statistic is particularly concerning given that the global prevalence of obesity has doubled since 1990 in adults and quadrupled in adolescents [9]. Initially perceived as an issue predominantly affecting high-income nations, obesity has, in recent years, emerged as a significant concern in low- and middle-income countries, including in both urban and underserved regions. The trend is not limited to adults; there has been a noticeable rise in obesity rates among children and adolescents. In Africa, the prevalence of obesity in children under five years old has surged by 23% since the year 2000, with nearly half of the children under five in Asia being overweight or obese [9]. Among children and adolescents aged 5–19, obesity rates climbed from 8% in 1990 to 20% in 2022 [9]. For adults aged 20 years old and older, the prevalence of obesity and severe obesity increased from 25.4 and 3.9 to 41.9 and 11.5, respectively, between the years of 1988–1994 and 2017–2018 [10]. As of 2017–2018, the prevalence is highest in the 40–59 age group (44.8), followed by individuals aged 60 years or older and 20–39 (42.8 and 40.0, respectively) [10]. The prevalence was higher in males compared to females for the 20–39 and 40–59 age groups (40.3 vs. 39.7 and 46.4 vs. 43.3, respectively) but lower in the 60 and over age group (42.2 vs. 43.3) [10]. Per the National Institutes of Health (NIH) 2017–2018 data, the percentage of females with severe obesity is higher than men (11.5% vs. 6.9%) [11]. When taking race into consideration, the percentage was higher in non-Hispanic Black adults compared to non-Hispanic White and non-Hispanic Asian adults (49.6% vs. 42.2% and 17.4%, respectively). Severe obesity had also a higher percentage in non-Hispanic Black males compared to non-Hispanic White and non-Hispanic Adian adults (13.8% vs. 9.3% and 2.0%, respectively) [11]. In children aged 2–19, the percentage was the highest among non-Hispanic Black girls (29.1%) followed by Hispanic boys (28.1%), Hispanic girls (23.0%) and non-Hispanic Black boys (19.4%) compared to other race groups [11]. The percentage was higher in non-Hispanic White boys compared to non-Hispanic White girls (17.4% vs. 14.8%) and in non-Hispanic Asian boys compared to non-Hispanic Asian girls (12.4% vs. 5.1%) [11].

## 3. Financial and Emotional Impacts of Obesity

In 2021, Cawley et al. published a retrospective analysis evaluating the medical costs of obesity between the years 2001 and 2016 [12]. It was noted that obesity increased annual medical spending by USD 2505 for U.S. adults. It was also noted that this increase correlates with the class of obesity with it being the highest for class 3 obesity (233.5% increase) compared to class 2 and class 1 obesity (120.0% increase and 68.4% increase, respectively) [12]. At a national level, between the years 2001 and 2016, the total direct medical costs of obesity in adults have increased by more than double (USD 124.2 to 260.6 billion) [12]. Beyond the economic implications, obesity also imposes a significant emotional toll and socioeconomic difficulties for individuals. The stigma associated with obesity can exacerbate poor eating behaviors and diminish physical activity levels, potentially worsening the problem [13]. Furthermore, it has been observed that individuals suffering from obesity are more prone to postponing or entirely skipping medical appointments. This avoidance not only hampers their access to necessary healthcare but also reinforces the cycle of health issues related to obesity [13].

## 4. Indications

### 4.1. Criteria

Bariatric surgery is indicated for patients who have struggled with severe obesity and have not achieved satisfactory weight loss through diet, exercise or other non-surgical methods. In 2022, the American Society of Metabolic and Bariatric Surgery (ASMBS) and the International Federation for Surgery of Obesity and Metabolic Disorders (IFSO) published their updates to the 1991 National Institutes of Health guidelines for bariatric surgery [14]. Original eligibility criteria were set to be a BMI ≥ 40 kg/m^2^ with no comorbidities or a BMI between 35 and 40 kg/m^2^ in the presence of certain obesity-related comorbidities (hypertension, dyslipidemia, diabetes mellitus and severe sleep apnea) [15]. Now, despite the fact that BMI does not necessarily reflect metabolic health and the potential for misclassification, BMI remains the most commonly used variable to classify individuals with obesity. Due to the existing evidence to support the significant benefit of metabolic and bariatric surgery (MBS) in individuals with class 1 obesity (BMI < 35 kg/m^2^), metabolic surgery is currently considered for class 1 obesity. MBS is also recommended for individuals with a BMI of 35 kg/m^2^ or more, regardless of comorbidities [14].

### 4.2. Special Considerations

- Special considerations were made for the Asian population, and the threshold to offer bariatric surgery was adjusted to a BMI of 27.5 kg/m^2^ or more [14].
- The American Academy of Pediatrics and the ASMBS recommend consideration of MBS in children and adolescents with a BMI higher than 120% of the 95th percentile and major comorbidities or a BMI higher than 140% of the 95th percentile [14,16]. In this patient population, sleeve gastrectomy is the most performed operation due to the comparative weight loss results and desirable safety profile [16].
- SG might be the preferred surgical option for immunosuppressed patients. Hefler et al. evaluated 30-day outcomes for immunosuppressed patients undergoing SG and RYGB [17]. Although there was no statistically significant difference in mortality between SG and RYGB, RYGB was associated with higher risk of major complications (9.6% vs. 5%, *p* < 0.001), re-operation (3.4% vs. 1.3%, *p* < 0.001), re-intervention (3.7% vs. 1.6%, *p* < 0.001), re-admission (7.9% vs. 5.3%, *p* = 0.002) and deep SSI (0.8% vs. 0.1%, *p* = 0.003) [17].
- SG serves as an excellent option for weight loss in morbidly obese patients with gastrointestinal stromal tumors (GISTs), gastric neuroendocrine tumors or conditions that require endoscopic surveillance of the stomach [18].

### 4.3. Sleeve Gastrectomy as a Bridge to Transplant

- The 2016 International Society for Heart Lung Transplantation listing criteria suggested that a pre-transplant BMI > 30 kg/m^2^ is associated with a poor outcome after cardiac transplantation, with an even worse outcome with a pre-transplant BMI > 35 kg/m^2^. Weight loss is recommended for this patient population before listing for cardiac transplantation [19]. Sleeve gastrectomy is thought to assist with weight loss and permit cardiac transplantation in patients with left ventricular assist devices (LVADs) [20,21]. Sharma et al. evaluated the role and safety of metabolic surgery in heart failure patients with LVADs awaiting heart transplants. In this systematic review, 271 patients were identified in the literature. Of these patients, 95.6% underwent sleeve gastrectomy. The most frequently reported complications were major adverse cardiovascular events (30.2%), followed by GI-related bleeding (20.9%) and LVAD pump thrombosis (11.6%). A total of 67 of the 271 patients (32.5%) received a heart transplant, with a mean time between bariatric surgery and heart transplant of 13.8 months [21]. Both LSG at the time of LVAD implantation and staged LSG following LVAD implantation have been reported [22]. There was no statistically significant difference between the two approaches in regard to the total incidence of postprocedural complications (16% and 23%, *p* = 0.82, for simultaneous vs. staged, respectively). There was no statistically significant difference in overall survival either (93% and 79%, *p* = 0.17, for simultaneous vs. staged, respectively) [22]. After SG, 66% were able to achieve listing requirements, and 33% received a heart transplant [22].
- The practice guidelines published by the American Association for the Study of Liver Diseases (AASLD) in 2013 indicated that severe obesity (BMI ≥ 40) is implicated in many adverse outcomes following liver transplantation [23].
- Bariatric surgery can aid in bridging morbidly obese patients with liver disease to liver transplantation (LT). SG is emerging as the preferred option for this patient population before or even after transplant [23,24].
- Metabolic surgery was found to improve access to kidney transplantation, enhance graft function and survival and increase kidney donation rate. SG has been associated with lower postoperative adverse outcomes and is preferred to RYGB, even in living donors, due to its low risk profile [25].

## 5. Our Technical Approach

**Figure 2:**
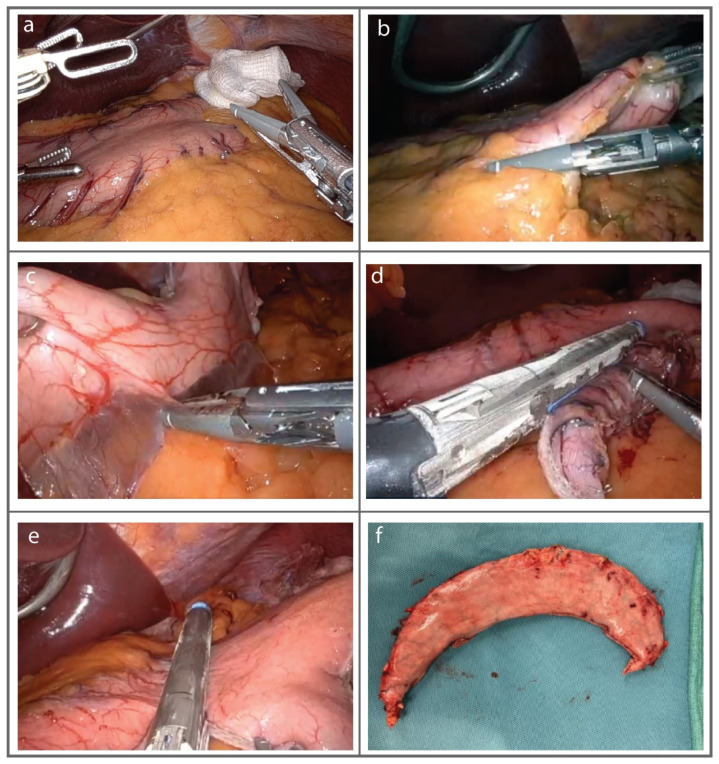
Surgical steps of sleeve gastrectomy. (**a**) Exposing pars flaccida, (**b**) Dividing the short gastric vessels, (**c**) Mobilizing the stomach posteriorly, (**d**) fashioning the sleeve with staples, (**e**) Transecting the gastric fundus, (**f**) Resected gastric specimen.

- Patient Positioning: The patient is placed in the supine position with their arms out. Footboard placement and securement of the legs are recommended to allow safe position manipulation intraoperatively. Two monitors are placed parallel to the patient’s shoulders. We do not typically place a Foley catheter in laparoscopic or robotic sleeve gastrectomies.
- Trocar Placement: Pneumoperitoneum is established through the Veress needle technique at Palmer’s point in the LUQ. Entrance into the peritoneal cavity is normally obtained with a 12 mm optical trocar in the supraumbilical region. An additional two 5 mm trocars are placed in the left upper quadrant and lateral flank. The Nathanson retractor is fashioned to gently retract the liver cephalad and expose pars flaccida (Figure 2a), and an additional 12 mm trocar is placed in the right upper quadrant.
- Gastric Dissection: Initial inspection is performed of the liver and of the hiatus to evaluate for hiatal hernia. The short gastric vessels are then divided with a bipolar energy device (Figure 2b), starting mid-body and moving up towards the left crus of the diaphragm, with full mobilization of the stomach posteriorly as well (Figure 2c). The dissection is then taken down towards the pylorus. Sometimes, additional 5 mm ports in the RUQ and LUQ can be helpful to prevent excessive torching if the dissection challenging due to limited space or body habitus.
- Fashioning the Sleeve: Approximately 4 cm proximal to the pylorus, a sleeve is constructed after a 40 French bougie is inserted. Sequential firings of the endo GIA stapler using purple loads with staple reinforcement are used to create a sleeve gastrectomy following the lesser curve of the stomach (Figure 2d). We are always careful not to encroach on the esophagus when the fundus is removed (Figure 2e). Sometimes, clips are needed to ensure hemostasis. The specimen is then extracted through the 12 mm trocar after a figure-of-eight #0 Vicryl suture is placed to close the fascial defect (Figure 2f).

## 6. Technical Considerations

### 6.1. Starting the Dissection

It is advised that the resection distance from the pylorus (DFP) is at least 3 cm in length. Although a shorter distance (3 cm or less) has been associated with a higher % EWL at 24 m, it has also been associated with a statistically significant increase in nausea and emesis in the early postoperative period (30 days) [26]. In a meta-analysis of randomized controlled trials by Diab et al., prolonged nausea and emesis (for more than 3–4 weeks) were also noted in the 2 cm DFP group. The study could not establish a difference in the incidence of postprocedural bleeding and leak. The incidence of GERD in the 6 cm DFP group was lower at the 6-month mark; however, this was not sustained at the 24-month mark. There was, however, a statistically significant difference in weight loss between the two groups, with a higher percentage of total weight loss in the 2 cm group [27].

### 6.2. Staple Size

Hajeychia et al. conducted a prospective ex vivo study evaluating leak pressures of different staple sizes (black endo GIA with a closing range of 1.75–2.25 mm, purple endo GIA with a closing range of 1.25–1.75 mm and tan/vascular endo GIA with a closing range of 0.75–1.25 mm) in the extracted portion of the stomach of 15 patients [28]. The black load was used to staple the distal and middle portion of the sleeve, and the purple load was used for the proximal part near the angle of His. A Veress needle was used to insufflate the stomach during the leak test. After testing the specimen, a new staple line was formed using the tan/vascular load just below the purple staple line to replace it for additional testing. No leaks occurred in the black staple line. When comparing the tan/vascular staple line to the purple staple line, it was noted that the first had a higher leak pressure of 28.9 ± 1.06 mmHg compared to the purple staple line, which had a pressure of 14.9 + 1.28 mmHg [28]. Prospective clinical trials are recommended to further evaluate the effect of staple size on leak rate.

### 6.3. Staple Line Reinforcement

In a large retrospective cohort including 346,530 patients by Highet et al., reinforced stapling and non-reinforced stapling were compared [29]. Re-enforcement techniques included stapling reinforcement, oversewing or both. Reinforced stapling was associated with a statistically significant decreased rate of minor bleed (0.45% vs. 0.59%, *p* < 0.0001, for reinforced vs. non-enforced, respectively). There was no statistically significant difference in major bleed (0.05% for both arms, *p* = 0.8841) or leaks (0.24% vs. 0.26%, *p* = 0.4812, for reinforced vs. non-reinforced, respectively). When looking at cost, they failed to establish the cost-effectiveness of stapler reinforcement, as the resultant base-case incremental cost-effectiveness ratio (ICER) was USD 40,553,000, which is significantly higher than the USD 150,000 willingness-to-pay threshold [29].

### 6.4. Angle of His

Kleidi et al. evaluated the effect of the proximity of transection line around the angle of His on postoperative gastroesophageal reflux disease [30]. This was a prospective cohort that included 23 patients with no prior history of GERD or dysmotility. A Likert scale score was used to capture the severity of GERD. It was noted that there was a statistically significant increase in heartburn, dysphagia and total score following SG. Interestingly, the presence of esophageal tissue on histopathology of the resected stomach correlated with a statistically significant increase in residual lower esophageal sphincter pressure, total GERD score and more severe heartburn [30]. Extra caution is advised to prevent over-approximation of the angle of His when transecting the sleeve proximally.

### 6.5. Intraoperative Adjuncts

The use of indocyanine green (ICG) to assess hemostasis and evaluate for leaks after fashioning the sleeve has been reported [31]. In the study conducted by Pavone et al., the application of ICG imaging technology was explored in 82 patients undergoing LSG. The effectiveness of ICG was determined through its ability to confirm adequate gastric perfusion, characterized by the direct and distinct visibility of fluorescence around the gastric tube within 150 to 180 s following intravenous administration. Despite the promising results, the study noted a minor limitation with a failure rate of 1.2% in detecting gastric leaks using ICG imaging. This underscores the technology’s overall promising efficacy in enhancing surgical outcomes [31]. More prospective data are needed to further evaluate this technology’s utility and effectiveness.

In their study, Chen et al. highlighted the value of intraoperative endoscopy (IOE) in identifying potential complications during LSG, such as stenosis or leaks. Remarkably, their findings indicated that while no leaks were detected using IOE, there was a small incidence (0.9% or 3 out of 352 patients) of gastric stenosis, which was promptly rectified during the operation. The significance of IOE became particularly evident when comparing outcomes between novice and experienced surgeons; gastric stenosis was identified exclusively in surgeries performed by less experienced surgeons (defined as surgeons who have performed 1–30 SG), with a notable difference in detection rates (0% among the more experienced group, *p* = 0.003). This underscores the importance of IOE as a valuable diagnostic tool, especially for surgeons in the early stages of their practice, enhancing patient safety and surgical efficacy [32].

## 7. Postoperative Care

- In our institution, postoperative patient care is meticulously designed to ensure symptomatic relief, including as-needed antinausea medications and multimodal pain control. We aim to closely monitor and encourage oral intake. The dietary protocol is specifically tailored to support early recovery, beginning with clear liquids on postoperative day zero. This is a crucial first step in the patient’s post-surgery diet, aiming to keep the body hydrated without straining the digestive system. As the patient’s tolerance improves, the diet is gradually advanced to full liquids by postoperative day one. This progression is carefully monitored to ensure the patient’s comfort and to adjust to their individual recovery pace.
- To further support nutritional intake and wound healing, supplemental protein shakes are incorporated into the patient’s diet three times daily, alongside meals. A dietitian meets with the patients on postoperative day one to ensure a proper understanding of their nutritional plan perioperatively and after discharge.
- Given the increased risk of blood clots following surgery, we implement chemoprophylaxis as a preventive measure against deep venous thrombosis (DVT). The timing of its initiation is carefully considered on a case-by-case basis to balance the benefits of early thrombosis prevention with the risk of bleeding. In routine cases, we prefer to start chemoprophylaxis on postoperative day zero.
- However, in patients presenting with baseline coagulopathies or those undergoing complex, re-operative surgeries with challenging dissections, a more cautious approach is adopted. For these patients, the initiation of chemoprophylaxis may be delayed until postoperative day one.
- The need for extended thromboprophylaxis on discharge is determined based on the Aminian et al. risk assessment tool [33].
- Patients are typically discharged on postoperative day one or two, following a thorough assessment to ensure they can tolerate oral intake effectively and their symptoms are well managed to be prepared to continue their recovery at home. Discharge criteria also include the confirmation that their laboratory results and vital signs are within satisfactory ranges, indicating a stable and positive trajectory.

In a randomized controlled trial by Papasavas et al., an Enhanced Recovery After Surgery (ERAS) protocol showed significant benefits over traditional care [34]. This innovative approach integrates comprehensive measures, including pre-admission nutritional support emphasizing a glycogen-depleted diet, pre-surgical hydration, deep venous thrombosis prevention and a multimodal regimen for pain and nausea control that begins during surgery and extends into the postoperative period.

The implementation of ERAS led to a remarkable reduction in opioid consumption on the hospital ward, with figures dropping to 72.3% from 95.4% compared to standard care. This decline was evident in both oral (58.5% from 80.0%, *p* = 0.008) and injectable narcotics (55.4% from 93.8%, *p* < 0.001), alongside a decrease in morphine milligram equivalent (MME) usage to 18.0 from 28.8 (*p* = 0.028). Additionally, patients under the ERAS protocol experienced shorter hospital stays, with the total duration reduced to 49.98 h from 52.15 h (*p* = 0.008) and the functional length of stay significantly lowered to 27.97 h from 44.37 h (*p* = 0.001). This study underscores the efficacy of ERAS in enhancing postoperative recovery, reducing opioid reliance and expediting hospital discharge [35].

## 8. Outcomes and Complications

### 8.1. Weight Loss

The percentage of excess weight loss (%EWL) after bariatric surgery is a commonly used measure of the surgery’s success and the patient’s progress toward achieving a healthier weight. %EWL is calculated by comparing the patient’s weight loss to their excess weight, which is the difference between their pre-surgery weight and their ideal weight, based on standard body mass index (BMI) charts. However, the %EWL can vary based on the definitions of ideal body weight (IBW) used and the pre-operative weight. Ideal weight is based on a BMI considered healthy, often set at a BMI of 25 kg/m^2^ [36,37].

In 2013, Gagner et al. published a survey on LSG at the Fourth International Consensus Summit (2012). The survey captured a total of 46,133 LSGs performed by 130 surgeons. They reported a mean %EWL at year 1 of 59.3%; year 2, 59.0%; year 3, 54.7%; year 4, 52.3%; year 5, 52.4%; and year 6, 50.6% [38].

A number of clinical trials with long-term follow-up have been published including, but not limited to, the SLEEVEPASS trial, which is one of the largest clinical trials evaluating long-term outcomes of sleeve gastrectomy (SG) vs. Roux-en-Y gastric bypass (RYGB). This multicenter trial enrolled 240 patients with morbid obesity aged 18–60 who were randomized to SG or RYGB and followed for 10 years [39]. At 5 years, there was no statistically significant difference between the two groups in percentage excess weight loss (%EWL): 49% (95% CI, 45–52%) after sleeve gastrectomy and 57% (95% CI, 53–61%) after gastric bypass [39]. The SM-BOSS trial also compared the effect of SG vs. RYGB on weight loss in patients with morbid obesity. Mean weight loss as a percentage was lower at 5 years in SG (SG, 25.0% vs. RYGB, 28.6%; 95% CI, −6.7–0.6%). Although both SG and RYGB achieved satisfactory weight loss results, there was no statistically significant difference in BMI at 5 years between the two procedures (95% CI, 0.77–2.6), and mean weight reduction was not significantly different between the two groups at 5 years (SG, 33.0 kg vs. RYGB, 36.6 kg; 95% CI, −1.8–9.0 kg) [40].

However, based on the SLEEVEPASS trial findings at 10 years, the two procedures were not equivalent for weight loss. The estimated mean %EWL was 43.5% (95% CI, 39.8–47.2) after SG and 51.9% (95% CI, 48.1–55.6) after RYGB. The model-based estimate of mean %EWL was 8.4 percentage points (95% CI, 3.1–13.6) higher after RYGB [41].

In a systematic review and meta-analysis conducted by Yeung et al., the safety and efficacy of sleeve gastrectomy (SG) were thoroughly evaluated. The study highlighted an average excess weight loss (%EWL) of 62%, demonstrating SG’s effectiveness in significant weight reduction. Additionally, the analysis showed a remarkable body mass index (BMI) reduction of −13.29 kg/m^2^, sustainable in the long term (results closely aligning at −12.56 kg/m^2^ at 24 months or longer) [42].

### 8.2. Esophagitis and Gastroesophageal Reflux Disease

The Osberg Reflux Working Group data were published in 2021. In this study, they aimed to compare the 1-year effect of SG and RYGB on secondary GERD outcomes. This trial included 109 patients with 1-year follow-up. Twenty-nine percent of these patients had GERD symptoms and fifty-eight percent had erosive gastritis pre-operatively. At 1 year, the SG group had a higher prevalence of GERD symptoms measured by the Gastrointestinal Symptoms Rating Scale—Reflux (score equals or greater than 20) compared to RYGB (17% vs. 6% (*p* = 0.070)). This difference was not statistically significant. Similar findings were noted when captured by the Gastroesophageal Reflux Disease Questionnaire Score (score equal to or greater than 8) (13% vs. 2% (*p* = 0.026)). Erosive gastritis was noted in 48% of SG patients compared to 33% of RYGB patients (*p* = 0.14); this difference was also not statistically significant. However, SG was associated with a statistically significant elevated risk of pathologic acid reflux (DeMeester score of 14.72 or higher) (21% vs. 7% for SG and RYGB, respectively, *p* < 0.001) [43]. 

Similar to the Oseberg trial findings, the SLEEVEPASS trial noted that the prevalence of esophagitis was higher after SG than RYGB (49% vs. 16%, respectively (*p* < 0.001)). However, there was no statistically significant difference in Barrett’s esophagus between the two groups [41].

The SM-BOSS trial reported statistically significant elevated GERD remission rates in the RYGB group compared to the SG group (60.4% vs. 25% (*p* = 0.002)). Its data also conveyed worsened GERD symptoms in the SG group compared to the RYGB group (31.8% vs. 6.3% (*p* = 0.006)) [40].

A systematic review and meta-analysis by Yeung et al. reflected significantly elevated rates of upper gastrointestinal tract pathologies following SG. The analysis revealed a 19% overall increase in reported reflux and a 23% increase in de novo reflux following surgery. Furthermore, the prevalence of esophagitis reached 6%, escalating to 8% in the long term (>24 m postoperatively). The incidence of postoperative hiatal hernias was found to be high, at 41%. Additionally, the utilization of proton pump inhibitors (PPIs) stood at 38%; however, only 4% of patients required a revision to RYGB due to refractory reflux disease, highlighting the critical need for careful patient selection [42].

### 8.3. Resolution of Comorbidities

The Osberg trial showed the RYGB group to have statistically significantly higher type 2 diabetes remission rates compared to the SG group (relative risk (RR) 1.57, *p* = 0.005) [44].

In a Swedish multicenter randomized controlled trial published by Wallenius et al., the effect of metabolic bariatric surgery was studied in individuals with type 2 diabetes. Remission was not significantly different between the two groups. It was achieved in 44% and 46% (*p* = 0.897) at 1 year and 48% and 55% (*p* = 0.654) at 2 years of follow-up for SG and RYGB, respectively [45].

In the SLEEVEPASS trial, SG and RYGB were able to achieve DM remission in 26% and 33% of patients, respectively, with no statistically significant difference between the two groups. Similarly, there was no statistically significant difference in dyslipidemia remission; however, both operations were successful in achieving remission with normal lipid values and no medications in 19% after SG and in 35% after RYGB (*p* = 0.23). SG and RYGB have also been successful in reducing hypertension (HTN) medication use (32% vs. 24%, respectively) or even discontinuing HTN medications (8% vs. 24%, *p* = 0.04)). In regard to obstructive sleep apnea (OSA), 16% in the SG group vs. 31% in the RYGB group discontinued using CPAP (*p* = 0.30). The Moorehead–Ardelt QOL score was used to capture the quality of life outcomes. In both groups, the total QOL was significantly better at 10 years compared to the baseline [41].

The SM-BOSS trial reported a type 2 diabetes remission rate of 61.5% for the SG group. However, there was no statistically significant difference when compared to the RYGB group with a remission rate of 67.9 (*p* > 0.99). Similar findings were noted for dyslipidemia (42.6% vs. 62.3% (*p* = 0.09)), hypertension (62.5% vs. 70.3% (*p* > 0.99)) and obstructive sleep apnea (45.8% vs. 44.2% (*p* > 0.99)) [40].

In a retrospective review by Blanco et al., both SG and RYGB had a positive effect on cardiovascular health, with a reported atherosclerotic cardiovascular disease (ASCVD) 10-year SCORE absolute risk reduction of 3.9 ± 6.5% in SG patients and 2.9 ± 5.8% in RYGB patients (*p* = 0.3). They also reported a decrease in estimated heart age of 12.1 ± 15.6 years in SG versus 9.2 ± 9.6 years in RYGB (*p* = 0.1). No statistically significant difference was noted between the two groups [46].

### 8.4. Affect on Metabolic Syndrome

Per the International Diabetes Federation (IDF) guidelines, metabolic syndrome is defined by the presence of three out of five of the following parameters: visceral obesity measured by a waist circumference greater than or equal to 94 cm in males and 80 cm in females or a BMI > 30 kg/m^2^, hypertriglyceridemia (equal to or higher than 150 mg/dL), low HDL (<40 mg/dL in males and <50 mg/dL in females), HTN (equal to or higher than 130/85 mmHg) and fasting hyperglycemia (equal to or higher than 100 mg/dL) [47]. Hady et al. reported a reduction in the frequency of metabolic syndrome parameters after LSG [48]. The observed %EBL at 1 year was 61.03 ± 6.50%. Waist circumference decreased from 122.8 ± 18.4 cm to 89 ± 8.2 cm in females and in males from 134.2 ± 27.6 cm to 106 ± 9.66 cm. There was also a statistically significant decrease in insulin and glucose concentration, in addition to a positive effect on all components of the lipid profile and an improvement in hypertension. At the one-year mark, only 61/130 patients had met 3/5 of the criteria of metabolic syndrome [48].

### 8.5. Laparoscopic Sleeve Gastrectomy vs. Endoscopic Sleeve Gastrectomy

When discussing sleeve gastrectomy, it is imperative to compare laparoscopic sleeve gastrectomy (LSG) and endoscopic sleeve gastrectomy (ESG), the latter being suggested as a less invasive alternative gaining traction in the field. A systematic review and meta-analysis conducted by Marincola et al. shed light on the efficacy and safety of these two procedures [49]. The findings reveal that LSG offers superior weight loss outcomes, with an average percentage of excess weight loss (%EWL) of 80.32%, compared to 62.20% for ESG, marking a notable difference of 18.12% (*p* = 0.0001). However, when it comes to safety, both procedures demonstrate comparable profiles. The study reported a negligible difference in periprocedural complication rates—0.30% for LSG and 0.15% for ESG—with a difference of 0.15% (*p* = 0.2056), indicating no statistical significance [49].

### 8.6. Complications

In 2016, Alvarenga et al. published a large retrospective analysis evaluating the safety of SG as the primary treatment modality for morbid obesity. This large study included 1020 patients with an 8-year follow-up. Early (30-day) rates for leak and stricture were 0.1% for both. Late (>30-day) stricture rate was 0.49%. The overall conversion rates to RYGB were 0.43%, 0.52% and 0.31% due to failure, GERD or gastric outlet obstruction, respectively [50].

Both early (30-day) and late complications of SG and RYGB were captured in the SLEEVEPASS clinical trial. There was no statistically significant difference in minor or major complications between the two groups. Complications rates associated with SG were GERD (31.4%), ulceration or stricture (1.7%), dumping (0.8%), fistula and abscess (0.8%) and incisional hernia (2.5%) [41].

One of the most feared complications of SG is a staple line leak. Warner and Sasse documented a leak rate of 1.3% (*n* = 14/1070) following SG. All of those leaks occurred within 3 cm of the gastroesophageal junction on the proximal staple line and required either an endoscopic or surgical intervention. Certain technical considerations are thought to play a role in decreasing the leak rate, including the use of calibration tubing and avoiding tightening the sleeve around the tubing at the incisura. It is also advised to preserve the vascular branches supplying the cardia, especially the posterior branches, ensuring proper distancing between the linear stapler and the true gastroesophageal junction [51].

Rutte et al. evaluated the outcomes of SG using their institutional data in a retrospective analysis using prospectively collected registry data, published in 2014. This study included a total of 1041 patients with at least 1-year follow-up. They reported a leak rate of 2.3%, and 25% of these patients required laparoscopy, 33% were managed endoscopically with drainage or clip placement and 29% required radiologic drainage. The reported revision rate was 6.8% [52].

## 9. Revisional Surgery after Sleeve Gastrectomy

The most common indications for SG revision to RYGB are gastroesophageal reflux disease, weight recurrence and inadequate weight loss [53]. In a meta-analysis evaluating the long-term (7 or more years) outcomes of SG by Clapp et al., they reported a recidivism (<50% EWL) rate of 27.8% (95% CI: 22.8–32.7%) without any heterogeneity. The reported rates of revision due to weight regain or GERD were 13.1% (95% CI: 5.6–20.6%) and 2.9% (95% CI: 1.0–4.9%), respectively [54]. Using the MBSAQIP database, Dang et al. compared primary RYGB (P-RYGB) to sleeve gastrectomy with RYGB (SG-RYGB) to better understand the safety profile of this revisional metabolic procedure. It was noted that SG-RYGB has a higher risk of serious complications compared to P-RYGB (7.2% and 5%, respectively (*p* < 0.001)). SG-RYGB also carried a statistically significant elevated risk for postoperative bleeding (2% vs. 1.6%, *p* < 0.001), re-operation (3% vs. 1.9%, *p* < 1.001), re-admission (7.3% vs. 4.8%, *p* < 0.001), deep surgical site infection (1% vs. 0.5%, *p* < 0.001) and sepsis (0.3% vs. 0.1%, *p* < 0.001). However, there was no statistically significant difference in 30-day mortality (1% vs. 1%, *p* = 0.385) [53].

In a detailed systematic review and meta-analysis conducted by Dantas et al., the study focused on evaluating only anastomosis gastric bypass (OAGB) as a viable and safe option following unsuccessful sleeve gastrectomy (SG), primarily due to weight regain or insufficient weight loss. The time span between undergoing SG and conversion to OAGB was noted to range from 38.5 to 68.4 months. The analysis highlighted that OAGB led to superior total weight loss (TWL) outcomes when compared to Roux-en-Y gastric bypass (RYGB), with a mean difference of −5.89 (95% CI: −6.80 to −4.97, *p* < 0.01). Despite the absence of a statistically significant difference in overall complication rates between the two surgical methods, the researchers pointed out the possibility of heterogeneity bias in their findings. Furthermore, the study underscored the current lack of long-term nutritional data necessary to fully endorse the safety profile of OAGB, suggesting an area for future research [35].

Kermansaravi et al. recently published an expert-modified Delphi consensus regarding re-do surgery following sleeve gastrectomy. A total of 91.3% agreed that multidisciplinary team (MDT) evaluation should be performed in all revisional procedures after SG. Also, 95.6% recommended esophagogastroduodenoscopy (EGD) prior to proceeding with revision or conversion. In addition, 97.8% thought RYGB to be an acceptable option. On the other hand, the majority disagreed with biliopancreatic diversion/duodenal switch (BPD/DS), single-anastomosis duodenal ileal bypass with sleeve gastrectomy (SADI-S) and single-anastomosis sleeve ileal bypass (SASI) being acceptable options for sleeve gastrectomy revision (93.3%, 93.3% and 95.5%, respectively) [55].

## 10. Conclusions

It is clearly notable that the increase in obesity rates is not restricted to adults alone, and it is definitely a growing concern among younger populations. This trend is particularly troubling as it sets the stage for a myriad of health issues in the future. The escalating obesity epidemic underscores the urgent need for a comprehensive strategy to mitigate this growing healthcare crisis.

Overall, sleeve gastrectomy stands out as a reliable and effective surgical solution for obesity, particularly suited to adolescents and patients with complex medical needs, including those who are candidates for transplant or are immunocompromised. This procedure not only facilitates significant weight loss but also maintains a lower risk profile compared to other bariatric surgeries. Finetuning sleeve gastrectomy operations in the future may involve leveraging advances in surgical techniques and technologies to enhance precision and creating personalized patient care pathways based on meticulous pre-operative screening to optimize outcomes.
